# Case Report: Glans penile necrosis in a patient with SARS-CoV-2 and leprosy infection

**DOI:** 10.12688/f1000research.84355.2

**Published:** 2022-03-15

**Authors:** Jufriady Ismy, Said Alfin Khalilullah, Aditya Fajar Bahagianto

**Affiliations:** 1 Division of Urology, Department of Surgery, Dr. Zainoel Abidin General Hospital, Universitas Syiah Kuala, Banda Aceh, Aceh, 23230, Indonesia; 2 Division of Urology, Department of Surgery, Dr. Hasan Sadikin General Hospital, Universitas Padjajaran, Bandung, Indonesia

**Keywords:** case report; COVID-19; gland penile necrosis; leprosy; SARS-CoV-2

## Abstract

**Background:** Severe acute respiratory syndrome coronavirus 2 (SARS-CoV-2) infection was firstly identified in China and has been declared a global pandemic. Several serious extrapulmonary manifestations due to SARS-CoV-2 infection have also been reported and associated with hypercoagulability thrombotic vasculopathy. In addition, cases of *Mycobacterium-leprae* infection have also been known associated with blood coagulation abnormality.

**Methods:** Here, we report a 56-year-old male with coronavirus disease-19 (COVID-19) with concomitant leprosy infection with manifestation of glans penile necrosis, presented to the emergency department with acute penile pain. This case is unique because no occlusion blood flow to the penile was observed in the radiographic imaging. We described the potential pathophysiology in this case through a literature review.

**Results:** The patient received treatment according to the COVID-19 protocol and was given low molecular weight heparin (LMWH) therapy for 4 days. During the follow up, the clinical and functional condition of the penis showed significant improvement.

**Conclusions:** Microthrombus involvement, platelet abnormalities and impaired hemostasis due to SARS-CoV-2 and leprosy co-infection are the hypothesis in this case report.

## Introduction

At the beginning of 2020, the World Health Organization declared novel coronavirus disease-2019 (COVID-19) infection as a pandemic which caused by severe acute respiratory syndrome coronavirus (SARS-CoV-2) that has led to a global health crisis due to acute respiratory distress syndrome (ARDS). ^1^ Most cases have mild-to-moderate symptoms, with approximately 15% developing severe pneumonia, while about 5% developing ARDS and organ failure. ^2^ Other serious complications related to SARS-CoV-19 infection include hypercoagulability and thrombotic vasculopathy with clinical manifestations such as coronary syndrome, deep vein thrombosis, ischemic stroke, and pulmonary embolism. ^3^^,^^4^ Recently, other serious clinical manifestations associated with genitalia condition were reported in patients with SARS-CoV-2 infection with penile ischemia. ^5^ In patients infected by SARS-CoV-2, penile priapism has also been reported. ^6^^,^^7^ Some authors have speculated that these conditions related to COVID-19 may be due to severe hypercoagulability and thrombotic tendency observed in patients with COVID-19. ^5^^,^^8^

On the other hand, penile involvement in leprosy is uncommon. ^9^ Leprosy (also known as Hansen’s disease) is an infection caused by *Mycobacterium leprae (M. leprae)* that can damage the peripheral nerves and bone absorption. Leprosy has two distinct phases: direct infection of macrophages and Schwann cells and reactional episodes. Approximately 50% of patients with leprosy are affected by a reactional episode, ^10^ which occurs because of endothelial inflammation leading to necrotizing pan-vasculitis. This condition, in severe cases, progresses to necrotic hemorrhagic lesions of the extremities and trunk, as well as Lucio’s phenomenon. ^11^^,^^12^

Penile ischemia or necrosis is rare due to its abundant blood circulations. Usually, penile necrosis is associated with thrombotic phenomena and calcium deposits in patients on dialysis. ^13^ However, no previous cases of penile ischemia in a COVID-19 patient who concomitantly infected with leprosy have ever been reported. Here, we presented a unique case of penile necrosis in acute COVID-19 and leprosy infection following the CARE guidelines. ^14^ To the best of our knowledge, this case is the first case presenting penile glans necrosis in COVID-19 patient concomitant leprosy. We also described the potential pathophysiology of glans penis necrosis in this case through a literature review.

## Case report

A 56-year-old Indonesian male patient was admitted to Dr. Zainoel Abidin General Hospital in Aceh, Indonesia. He was referred from a regional hospital with SARS-CoV-2 infection and necrotic penile glans. Complaints of changes in the color of the glans penis occur progressively without any past intervention. In addition, he also complained of pain in the penis, dysuria and difficulty urinating. He worked as a farmer, actively smoked since he was an adolescent, and does not consume any drugs or alcohol. Currently, he is undergoing treatment for leprosy disease. Table 1 summarizes the patient’s characteristics.

**Table 1.** Baseline characteristics of the patient.

	Patient characteristics
Positive *M. leprae* Infection (Skin biopsy)	April 03, 2021
Positive SARS-CoV-2 (RT-PCR)	June 22, 2021
Days between SARS-CoV-2 diagnosis and penile gland necrosis	5 days
Comorbidities	Hypertension for 3 years (under medication)
Past medication	Amlodipine 10 mg, Clofazimine 50 mg, Dapsone 100 mg
Laboratory test results at first presenting	White cells count 13.2 × 10 ^9^/L Hemoglobin 11.5 g/dl Platelet 310 × 10 ^9^/L Sodium 142 mmol/L Potassium 3.8 mmol/L Chloride 113 mmol/L Urea 7 mmol/L Creatinine 72 μmol/L Ferritin 25 μg/dL Prothrombin 67.4% Activated partial thromboplastin time 1.20% Fibrinogen 102 U/L D-Dimer 9530 ng/mL

SARS-CoV-2, Severe Acute Respiratory Syndrome Coronavirus 2; RT-PCR, Reverse transcription polymerase chain reaction.

The patient has been diagnosed with leprosy since April 2021, he complained of a rash and atrophy of the hand muscles. Then the patient received leprosy treatment with a regiment of clofazimine 50 mg and Dapsone 100 mg.

This patient had a past medical history of hypertension, which is well-controlled with 10 mg/day oral amlodipine. There was no complaint in micturition, meatal discharge, and other genital problems.

On examination, his condition was stable with blood pressure 134/91 mmHg, pulse 90 bpm, respiratory 22 breaths/minute, and oxygen saturation at 97% on room temperature. The patient was afebrile and fully alert. His localized status found a discoloration involving the penile glans ( Figure A).

**Figure A.**
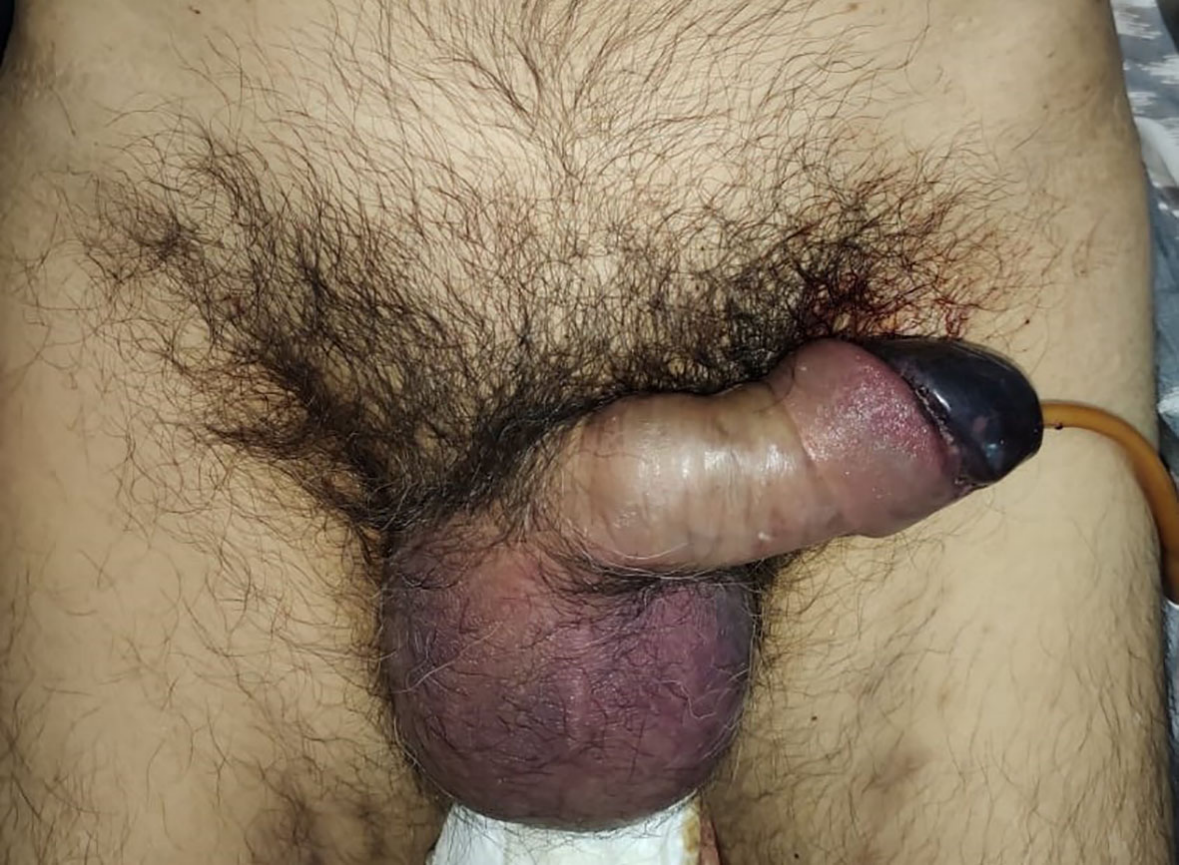
Color change at the glans penile, suggesting clinical signs of penile glans necrosis.

An urgent radiologic investigation with Doppler ultrasound of the penile and pelvic computerized tomography (CT) angiography revealed no occlusion nor thrombus on the arteria that supplies the penile ( Figure B).

**Figure B.**
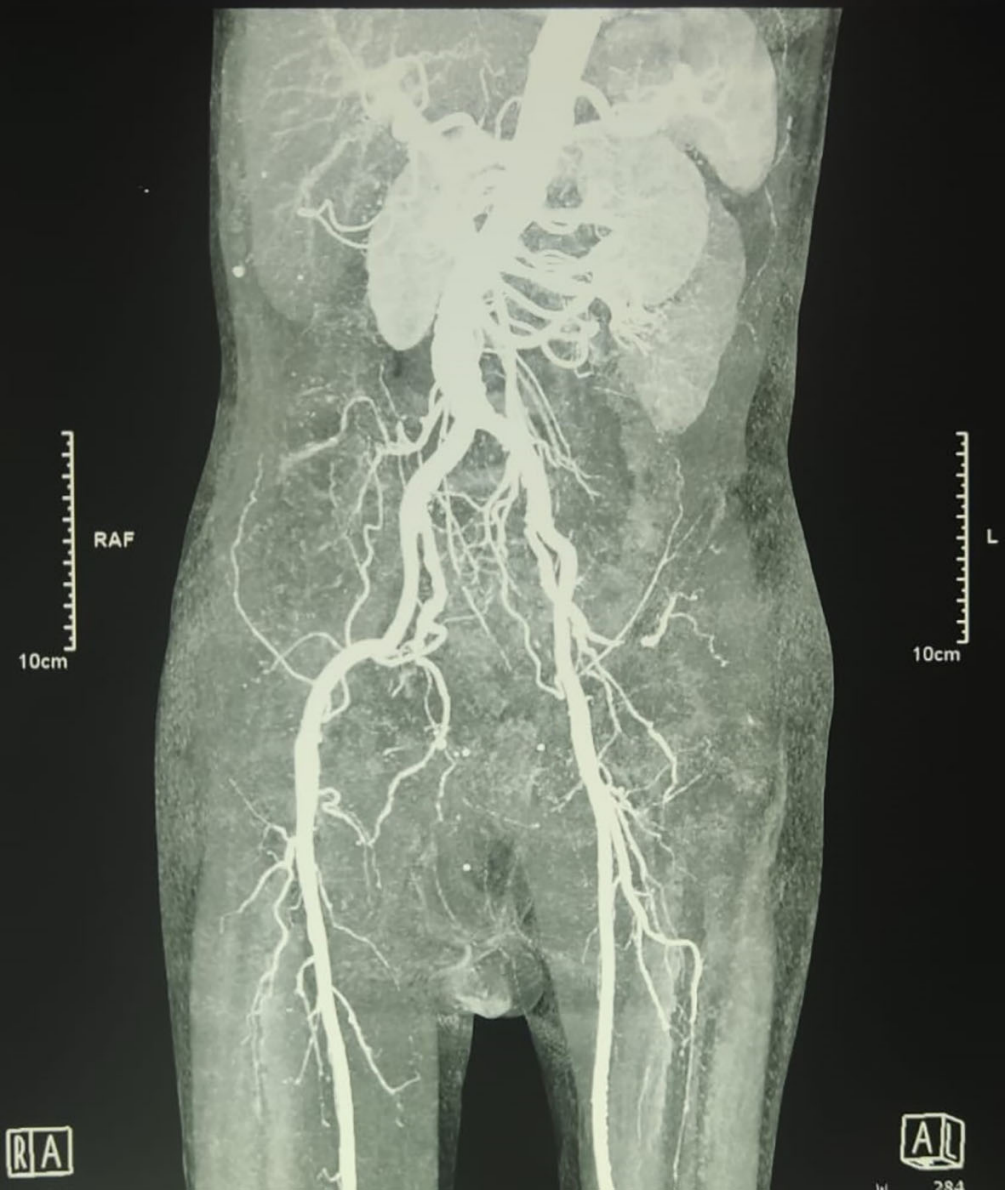
Pelvic Computerized Tomography Angiography showed no occlusion that obstructed blood flow in the right and left internal pudendal arteries was observed.

The patient was admitted to the COVID ward with therapy as follows; 2 L/min nasal oxygen, 1000 mg ceftriaxone injection twice daily, 10 mg amlodipine daily, 50mg clofazimine daily, 100mg dapsone daily, 5000 U vitamin D daily, 1000 mg vitamin C daily and 300 international unit low molecular weight heparin (LMWH) intravenous drip daily for 4 days. No challenges were found during treatment.

After 4 days of LMWH administration without any treatment changes, the clinical condition of the gland penile and laboratory of hemostatic was improved, the necrosis was less extensive and limited to one-third of the superficial of the glans penis ( Figure C). The patient then continued to isolate in the COVID-19 ward. On 6 weeks follow-up, the clinical and functional condition of the penis were excellent without any unexpected events. The patient’s urinary and erectile functions remained unchanged. The patient was also satisfied with the treatment that he received.

**Figure C.**
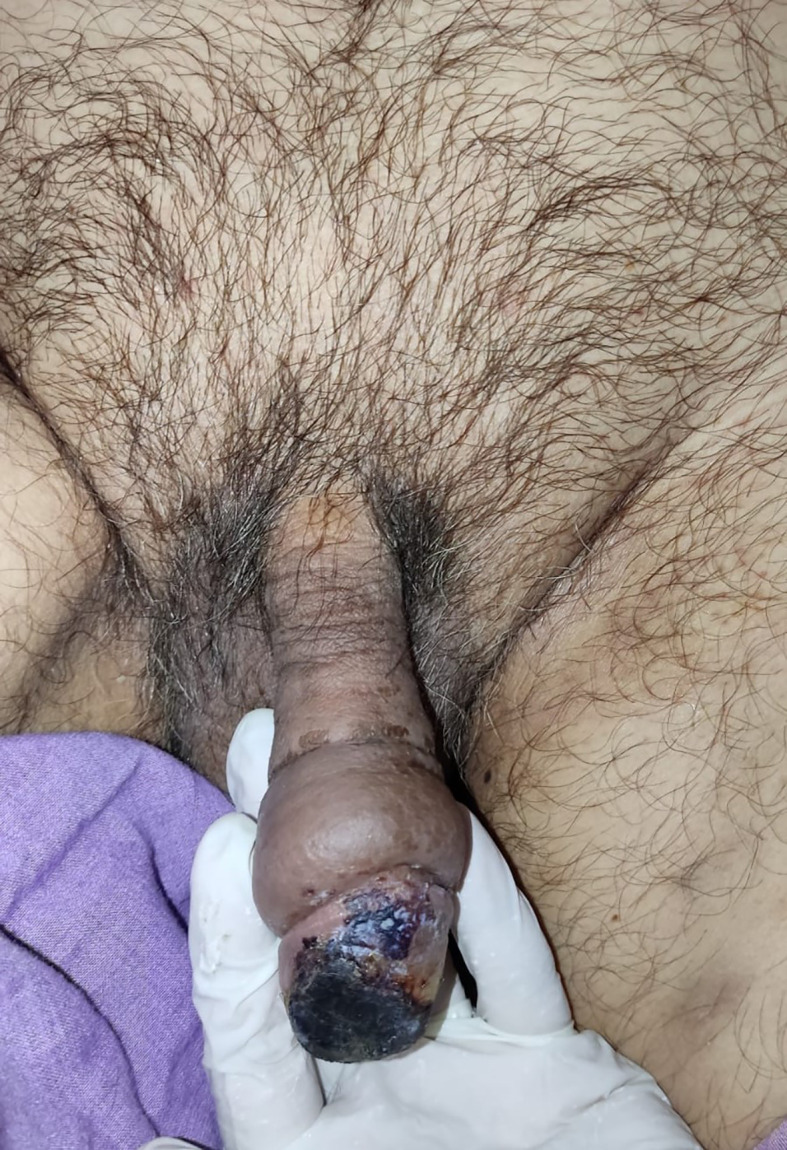
The clinical condition at 2-week follow-up after administration of low molecular weight heparin, the necrosis was less extensive and limited to one-third of the superficial of the glans penis.

## Discussion

We searched four online databases (the Medline EMBASE, Google Scholar, PubMed, and Scopus) for the literature review. Four terms related to the patient’s condition were combined in the searching strategy; 1) Penile; 2) Penis; 3) COVID-19 and 4) SARS-CoV-2 to investigate the relationship between COVID-19 and leprosy infection in necrosis of glans penis. In the end, we found only one publication by Sarkis *et al.* that presented the penile ischemic condition in COVID-19 infection. ^5^ This publication reported glans penile discoloration due to ischemia secondary to COVID-19. However, the patient had several comorbidities such as type 2 diabetes, hypertension, and end-stage kidney disease which could also be risk factors for thrombus vasculopathy.

Penile necrosis is linked to thrombotic events and calcium deposits in dialysis patients. ^13^ Another study also reported penile necrosis secondary to purpura fulminans. ^15^ One study in Japan described fifteen patients with penile necrosis due to calciphylaxis and a long history of diabetes. ^16^ In this condition, it is clearly understood that diabetic vasculopathy and calcium deposits can become a thrombus which causes impaired blood flow to penis. In our patient, he had no comorbid precipitating thrombus vasculopathy. Although the patient is an active smoker, we still cannot conclude that this is the cause of penile necrosis. Uniquely, the CT angiography investigation of our patient did not reveal any occlusion or thrombus.

On the basis of the above finding, we hypothesized that the patient’s penis glans necrosis is caused by COVID-19 and leprosy co-infection. Many studies have published the role of microvascular thrombus in SARS-CoV-2 infection. ^17^^–^^20^ There is a definite link between inflammation, hypercoagulation, and thrombosis, according to previous research. Part of this interaction is likely mediated by a cytokine storm, which increases the risk of developing disseminated intravascular coagulopathy. ^21^ The stimulation of coagulation pathways throughout the body by mediators produced during a cytokine storm can result in a prothrombotic condition marked by the formation of microthrombi, diffuse capillary blockage, tissue ischemia, and organ damage. ^18^ This pathogenesis indicates the possible tendency for physiopathology of glans penile necrosis secondary to SARS-CoV-2 infection.

Similar to SARS-CoV-2 infection, leprosy has long been known to trigger hemostasis disorders due to platelet abnormalities, blood coagulation, and fibrinolysis. ^10^ Several studies have previously reported that patients with tuberculoid leprosy who develop erythema nodosum leprosum (ENL) had a longer activated partial thromboplastin time (aPTT) with elevated fibrinogen and platelet activation. ^12^^,^^22^^,^^23^ One review of related studies conducted by Nery has described thrombophlebitis and pulmonary embolism in leprosy patients who develop ENL. ^24^ Another study also found that leprosy disease was associated with coronary thrombosis and cerebral vascular accidents. ^25^^,^^26^ These findings indicate that the severity of leprosy potentially leads to occlusion of blood flow which eventually causes organ damage.

We assume that patients co-infected with SARS-CoV-2 and leprosy have a greater risk of complications, especially hemostasis disorders and thrombus vasculopathy, as in our patient who showed elevated of D-Dimer and fibrinogen levels. This case report and literature review provide preliminary evidence of the association between SARS-CoV-2 and leprosy infection in glans penile necrosis; however, further studies are warranted.

This case report and literature review is limited by its short-term follow-up period, however its strength is that as we directly observed the patient rather than taking his information retrospectively, this reduces any potential bias.

Finally, several highlights should be considered in managing glans penile necrosis in patients with COVID-19 infection. As previously described, penile necrosis can occur as a result of a vasculopathy thrombus. On the other hand, COVID-19 and leprosy infection can also induce coagulopathy and microvascular thrombus. Therefore, choosing anticoagulant therapy than immediate aggressive amputation of the organ is still a viable option. Administering anti-thrombus therapy is a less-intrusive treatment approach that physicians can consider, which gave a good result in our patient.

## Conclusions

In our perspective, microthrombus formation, diffuse capillary occlusion and tissue necrosis are the basis of the etiology penile glans necrosis in our patient. It is likely associated with SARS-CoV-2 and leprosy co-infection. This hypothesis makes this case an interesting report. In addition, from what we know at the time of writing this manuscript, this is the first case report of glans penile necrosis in a patient with SARS-CoV-2 and leprosy co-infection.

## Data availability

All data underlying the results are available as part of the article and no additional source data are required.

## Consent

Written informed consent was obtained from the patient for publication of the patient’s images and this case report.
