# Supplementary material for: Case Report: Glans penile necrosis in a patient with SARS-CoV-2 and leprosy infection
Source: F1000Res. 2024 Feb 5;11:142. Originally published 2022 Feb 3. [Version 4] doi: 10.12688/f1000research.84355.4 (PMC11372346; doi:10.12688/f1000research.84355.4)
Supplement: Peer review history

# Reviewer report: Reviewer response for version 4

**DOI:** 10.5256/f1000research.162133.r318893

**Published:** 2024-09-09

**Authors:** Amílcar Sabino Damazo

**Affiliations:** 1 Faculdade de Medicina, Universidade Federal de Mato Grosso, Universidade de Brasília, Cuiabá, Brazil

Introduction

 Correct South Korea in the sentence: "The pandemic started in the seafood market of Wuhan, China, and rapidly spreading to Thailand, Japan, south Korea, Singapore, Iran, Italy, Spain, United States, United Emirates Arab, and United Kingdom."

**Correct SARS-CoV-2 in the sentence:**"Other serious complications related to SARS-CoV-19 infection include hypercoagulability and thrombotic vasculopathy with clinical manifestations such as coronary syndrome, deep vein thrombosis, ischemic stroke, and pulmonary embolism."

**The term phase is wrong for leprosy. Please rephrase it** **:**"Leprosy has two distinct phases: direct infection of macrophages and Schwann cells and reactional episodes."

 Results:

**Did the patient had any signs of eritema nodosum?**

 I recommend that the authors consider making grammatical adjustments to enhance the text's scientific accuracy.

Are enough details provided of any physical examination and diagnostic tests, treatment given and outcomes?

Yes

Is the case presented with sufficient detail to be useful for other practitioners?

Yes

Is sufficient discussion included of the importance of the findings and their relevance to future understanding of disease processes, diagnosis or treatment?

Partly

Is the background of the case’s history and progression described in sufficient detail?

Yes

Reviewer Expertise:

Leprosy diagnosis

I confirm that I have read this submission and believe that I have an appropriate level of expertise to confirm that it is of an acceptable scientific standard, however I have significant reservations, as outlined above.

# Reviewer report: Reviewer response for version 4

**DOI:** 10.5256/f1000research.162133.r243966

**Published:** 2024-09-03

**Authors:** Serena Bianchi, Diana Torge

**Affiliations:** 1 University of L’Aquila, L’Aquila, Italy; 2 Department of Clinical Medicine, Public Health, Life and Environmental Sciences, University of L'Aquila, L'Aquila, Italy

I have no further comments. Well done.

Are enough details provided of any physical examination and diagnostic tests, treatment given and outcomes?

Yes

Is the case presented with sufficient detail to be useful for other practitioners?

Partly

Is sufficient discussion included of the importance of the findings and their relevance to future understanding of disease processes, diagnosis or treatment?

Yes

Is the background of the case’s history and progression described in sufficient detail?

Partly

Reviewer Expertise:

Microscopy, Anatomy, Morphology, Histology, Pathology

We confirm that we have read this submission and believe that we have an appropriate level of expertise to confirm that it is of an acceptable scientific standard.

# Reviewer report: Reviewer response for version 3

**DOI:** 10.5256/f1000research.132446.r224136

**Published:** 2024-01-24

**Authors:** Serena Bianchi, Diana Torge

**Affiliations:** 1 University of L’Aquila, L’Aquila, Italy; 2 Department of Clinical Medicine, Public Health, Life and Environmental Sciences, University of L'Aquila, L'Aquila, Italy

The aim of this manuscript is to present a unique case of penile necrosis in acute COVID-19 and leprosy infection following the CARE guidelines.

 Overall, a significant effort was made to prepare this manuscript, which is quite interesting and unique. This article is impressive for the reviewer and audience of the scientific community as well as the study of SARS-CoV-2 infection. This manuscript in fact would show a significant impact,  rich content, providing a deep insight for some works: the study is within the journal’s scope, and I found it to be well-written, providing sufficient information. Even if the manuscript provides an organic overview, with a densely organized structure and based on well-synthetized evidence, there are several recommendations that can be implemented to improve the clarity and organization of the content. In fact, there are areas that could benefit from improvement to enhance the overall quality of the article. For these reasons, the manuscript requires major changes.

 Please find below an enumerated list of comments on my review of the manuscript:

 MINOR POINTS:

 The authors should provide a list of the abbreviations, mentioned in this manuscript.

 World Health Organization (WHO): in this introductive sentence, please, add the related abbreviation.

 MAJOR POINTS:

 INTRODUCTION:

 In the introductive section of this manuscript, there is a lack of evidence regarding the temporal background  and context of SARS-CoV-2 infection: in fact, the pandemic started in the seafood market of Wuhan, China, in December 2019, rapidly spreading to Thailand, Japan, South Korea, Singapore, Iran, Italy, Spain, the USA, the UAE, and the U.K. Moreover, on 11 March 2020 the World Health Organization (WHO) declared the outbreak of COVID-19 disease a pandemic (Umakanthan S. et al., (2020) ^1^). This will provide a  clear background and context, ensuring that readers understand the temporal dynamics of SARS-CoV-2 infection.

 Another major point of this manuscript is the lack of evidence about the molecular features of SARS-CoV-2 infection: in fact, the causative agent for COVID-19, severe acute respiratory syndrome coronavirus-2 (SARS-CoV-2), is an enveloped positive single-stranded RNA virus, whose viral genome includes a 5’ terminal, which encodes proteins essential for virus replication, and a 3’ terminal, that includes five structural proteins, Spike protein (S), membrane protein (M), nucleocapsid protein (N), envelope protein (E), and hemagglutinin-esterase protein (HE) (Lu R. et al., (2020) ^2^).

 Furthermore, endothelial damage, associated to COVID-19, is likely to compromise the penile vascular flow, also determining a significant impairment in the erectile function, as suggested by recent structural and ultrastructural studies. SARS-CoV-2 infection exerts its histopathological and clinical effects also on male reproductive tissues, probably due to the simultaneous expression of ACE and TMPRSS2 proteins, altered immunological and endothelial pathways. Specifically, the endothelial integrity is essential for erectile function, so  impairment in the erectile function can be considered an extra-pulmonary manifestation of SARS-CoV-2 infection (Torge D., et al., (2022) ^3^). Exploring morphological evidence associated to SARS-CoV-2 infection in the introduction, referencing recent articles, will enrich the manuscript's quality and captivate reader interest. It is advisable to incorporate pertinent literature quotes to strengthen the manuscript's credibility and relevance. This is the major concern of this manuscript.

 For these reasons, the authors should improve the references section, by including recent evidence on this topic.

 The main topic is interesting, and certainly of great clinical impact. As regards the originality and strengths of this manuscript, this is a significant contribute to the ongoing research on this topic, as it extends the research field on a unique case of penile necrosis in acute COVID-19 and leprosy infection. Overall, the contents are rich, and the authors also give their deep insight for some works.

 As regards the section of methods, there is a specific and detailed explanation for the methods used in this study: this is particularly significant, since the manuscript relies on a multitude of methodological and statistical analysis, to derive its conclusions. The methodology applied is overall correct, the results are reliable and adequately discussed.

 The conclusion of this manuscript is perfectly in line with the main purpose of the paper: the authors have designed and conducted the study properly. As regards the conclusions, they are well written and present an adequate balance between the description of previous findings and the results presented by the authors. The authors in fact provided a focused conclusion, which summarizes the key points and challenges discussed in the paper and offers insights into future research directions.

 Finally, this manuscript also shows a basic structure, properly divided and looks like very informative on this topic. Furthermore, figures and tables are complete, organized in an organic manner and easy to read.

 In conclusion, this manuscript is densely presented and well organized, based on well-synthetized evidence. The authors were lucid in their style of writing, making it easy to read and understand the message, portrayed in the manuscript. Besides, the methodology design was appropriately implemented within the study. However, many of the topics are very concisely covered. This manuscript provided a comprehensive analysis of current knowledge in this field. Moreover, this research has futuristic importance and could be potential for future research. However, major concerns of this manuscript are with the introductive section: for these reasons, I have major comments for this section, for improvement. The article is accurate and provides relevant information on the topic and I have some major points to make, that may help to improve the quality of the current manuscript and maximize its scientific impact. I would accept this manuscript if the comments are addressed properly.

Are enough details provided of any physical examination and diagnostic tests, treatment given and outcomes?

Yes

Is the case presented with sufficient detail to be useful for other practitioners?

Partly

Is sufficient discussion included of the importance of the findings and their relevance to future understanding of disease processes, diagnosis or treatment?

Yes

Is the background of the case’s history and progression described in sufficient detail?

Partly

Reviewer Expertise:

Microscopy, Anatomy, Morphology, Histology, Pathology

We confirm that we have read this submission and believe that we have an appropriate level of expertise to confirm that it is of an acceptable scientific standard, however we have significant reservations, as outlined above.

## References

[1] : Origin, transmission, diagnosis and management of coronavirus disease 2019 (COVID-19). *Postgrad Med J* .2020;96(1142) : 10.1136/postgradmedj-2020-138234 753-758 10.1136/postgradmedj-2020-138234 32563999 PMC10016932

[2] : Genomic characterisation and epidemiology of 2019 novel coronavirus: implications for virus origins and receptor binding. *Lancet* .2020;395(10224) : 10.1016/S0140-6736(20)30251-8 565-574 10.1016/S0140-6736(20)30251-8 32007145 PMC7159086

[3] : Histopathological Features of SARS-CoV-2 in Extrapulmonary Organ Infection: A Systematic Review of Literature. *Pathogens* .2022;11(8) : 10.3390/pathogens11080867 10.3390/pathogens11080867 36014988 PMC9415619

# Response

**Published:** 2024-01-31

**Authors:** Jufriady Ismy

**Affiliations:** Universitas Syiah Kuala, Indonesia

Thank you reviewer for the suggestions on this manuscript.

 We have improved this manuscript according to your suggestions, such as; updating abbreviations, adding references in the introduction such as the history of the SARS-CoV-2 pandemic, molecular features of SARS-CoV-2 infection and an explanation of endothelial damage associated to COVID-19.

# Reviewer report: Reviewer response for version 3

**DOI:** 10.5256/f1000research.132446.r135455

**Published:** 2022-04-25

**Authors:** Harrina Erlianti Rahardjo

**Affiliations:** 1 Department of Urology, Cipto Mangunkusumo Hospital-Faculty of Medicine, University of Indonesia, Jakarta, Indonesia

I have no further comments.

Are enough details provided of any physical examination and diagnostic tests, treatment given and outcomes?

Partly

Is the case presented with sufficient detail to be useful for other practitioners?

Partly

Is sufficient discussion included of the importance of the findings and their relevance to future understanding of disease processes, diagnosis or treatment?

Partly

Is the background of the case’s history and progression described in sufficient detail?

Yes

Reviewer Expertise:

Urology, functional urology, neurourology, urodynamic

I confirm that I have read this submission and believe that I have an appropriate level of expertise to confirm that it is of an acceptable scientific standard.

# Reviewer report: Reviewer response for version 2

**DOI:** 10.5256/f1000research.122365.r127619

**Published:** 2022-03-23

**Authors:** Harrina Erlianti Rahardjo

**Affiliations:** 1 Department of Urology, Cipto Mangunkusumo Hospital-Faculty of Medicine, University of Indonesia, Jakarta, Indonesia

Is there any clinical picture on 6 weeks follow up? It would be best to include this picture to show clearly the penile condition compared to week 2 and on admission. 

 How were the laboratory hemostatic parameters improved? Is there any data on hemostatic parameters on week 2 and week 6? It would be an important data alongside the clinical picture since one of the assumed pathophysiology is thrombosis.

Are enough details provided of any physical examination and diagnostic tests, treatment given and outcomes?

Partly

Is the case presented with sufficient detail to be useful for other practitioners?

Partly

Is sufficient discussion included of the importance of the findings and their relevance to future understanding of disease processes, diagnosis or treatment?

Partly

Is the background of the case’s history and progression described in sufficient detail?

Yes

Reviewer Expertise:

Urology, functional urology, neurourology, urodynamic

I confirm that I have read this submission and believe that I have an appropriate level of expertise to confirm that it is of an acceptable scientific standard, however I have significant reservations, as outlined above.

# Response

**Published:** 2022-04-18

**Authors:** Jufriady Ismy

**Affiliations:** Universitas Syiah Kuala, Indonesia

We Thank to the reviewer for their comments

 We apologize for not documenting the patient's clinical condition at 6 weeks of follow-up. After that time, the patient lost contact for follow-up.

 Laboratory data of homeostatic parameters after LWMH therapy was improved, we added the data of parameters laboratory after administration of LWMH in table 1. At week 6 of follow-up at the polyclinic, we did not check the laboratory haemostatic parameters again.

# Reviewer report: Reviewer response for version 1

**DOI:** 10.5256/f1000research.89378.r122238

**Published:** 2022-02-22

**Authors:** Harrina Erlianti Rahardjo

**Affiliations:** 1 Department of Urology, Cipto Mangunkusumo Hospital-Faculty of Medicine, University of Indonesia, Jakarta, Indonesia

This is a very interesting case report of penile necrosis in a patient with both a Covid and leprosy infection, perhaps this is the first case reported.

 However several details need to be provided. 

 In the case report the author has included the baseline data of the patient, however during treatment and follow up there was only a clinical picture after 4 days of treatment. The authors should also add the penile clinical condition on the 6th week follow up. The data is also lacking on the status of the patient's leprosy infection in terms of clinical history and medication. It would also add substantial information if the authors would include the changes in laboratory parameters throughout the course of treatment. Was it inline with the improvement seen clinically? Changes of the hemostasis lab parameters, clinical progress during treatment and perhaps cytokine data (if it was measured) would add up to a nice discussion.

Are enough details provided of any physical examination and diagnostic tests, treatment given and outcomes?

Partly

Is the case presented with sufficient detail to be useful for other practitioners?

Partly

Is sufficient discussion included of the importance of the findings and their relevance to future understanding of disease processes, diagnosis or treatment?

Partly

Is the background of the case’s history and progression described in sufficient detail?

Yes

Reviewer Expertise:

Urology, functional urology, neurourology, urodynamic

I confirm that I have read this submission and believe that I have an appropriate level of expertise to confirm that it is of an acceptable scientific standard, however I have significant reservations, as outlined above.

# Response

**Published:** 2022-03-11

**Authors:** Jufriady Ismy

**Affiliations:** Universitas Syiah Kuala, Indonesia

We thank to the reviewer for their comments,

 The clinical picture in this article is the clinical condition of the patient at 2 weeks of follow-up. we will improve the narrative in the article so that there are no misunderstandings. Meanwhile, we apologize for not documenting the patient's clinical condition at 6 weeks of follow-up.

 Regarding the history and medication of leprosy we have summarized it in table 1, but to add to the clinical history, we also include it in the article

 We apologize, because we do not have data related to cytokines, but changes in hemostasis laboratory parameters is in line with improving clinical conditions. We also added a revision of this information to the article.
